# Two new *Neohelicomyces* species (Tubeufiaceae, Tubeufiales) associated with *Coffea arabica* L. in Yunnan Province, China

**DOI:** 10.3897/mycokeys.127.173937

**Published:** 2026-01-29

**Authors:** Mei-Yan Han, Jing-Ya Yang, Samantha C. Karunarathna, Jaturong Kumla, Li Lu, De-Ge Zheng, Abdallah M. Elgorban, Alanoud T. Alfagham, Fu-Qiang Yu, Dong-Qin Dai, Li-Juan Zhang, Nakarin Suwannarach, Saowaluck Tibpromma

**Affiliations:** 1 Center for Yunnan Plateau Biological Resources Protection and Utilization & Yunnan International Joint Laboratory of Fungal Sustainable Utilization in South and Southeast Asia, College of Biology and Food Engineering, Qujing Normal University, Qujing 655099, China Bioengineering and Technological Research Center for Edible and Medicinal Fungi, Jiangxi Agricultural University Nanchang China; 2 Department of Biology, Faculty of Science, Chiang Mai University, Chiang Mai 50200, Thailand College of Biology and Food Engineering, Qujing Normal University Qujing China; 3 Center of Excellence in Microbial Diversity and Sustainable Utilization, Chiang Mai University, Chiang Mai 50200, Thailand Kunming Institute of Botany, Chinese Academy of Sciences Kunming China; 4 College of Agronomy and Biological Science, Yuxi Normal University, Yuxi, Yunnan, China Center of Excellence in Biotechnology Research Riyadh Saudi Arabia; 5 Office of Research Administration, Chiang Mai University, Chiang Mai 50200, Thailand College of Science, King Saud University Riyadh Saudi Arabia; 6 Bioengineering and Technological Research Center for Edible and Medicinal Fungi, Jiangxi Agricultural University, Nanchang 330045, China College of Agronomy and Biological Science, Yuxi Normal University Yuxi China; 7 Center of Excellence in Biotechnology Research (CEBR), King Saud University, Riyadh, Saudi Arabia Faculty of Science, Chiang Mai University Chiang Mai Thailand; 8 Department of Botany and Microbiology, College of Science, King Saud University, Riyadh, Saudi Arabia Center of Excellence in Microbial Diversity and Sustainable Utilization, Chiang Mai University Chiang Mai Thailand; 9 Germplasm Bank of Wild Species & Yunnan International Joint Laboratory of Fungal Sustainable Utilization in South and Southeast Asia, Kunming Institute of Botany, Chinese Academy of Sciences, Kunming 650201, China Office of Research Administration, Chiang Mai University Chiang Mai Thailand; 10 School of Food and Pharmaceutical Engineering, Guizhou Institute of Technology, Guiyang 550025, Guizhou, China School of Food and Pharmaceutical Engineering, Guizhou Institute of Technology Guiyang Thailand

**Keywords:** Coffee-associated microfungi, morphology, multigene phylogeny, new taxa, taxonomy

## Abstract

*Neohelicomyces* species comprise a group of helicosporous hyphomycetes, with over 90% of the currently accepted taxa reported from China, occurring in both freshwater and terrestrial habitats. Although the genus has been increasingly documented in recent years, its presence in crop-related ecosystems remains poorly understood, as most species have been reported from unknown hosts. In this study, a survey of fungi associated with *Coffea arabica* in Yunnan Province, China, was conducted, and fruiting bodies of helicosporous hyphomycetes were found on dead branches of coffee plants. Based on the morphological characteristics of conidiophores, conidiogenous cells, and conidia, in combination with multigene phylogenetic analyses (ITS, LSU, *rpb*2, and *tef*1-α), two novel species of *Neohelicomyces* (*N. coffeae* and *N. puerensis*) were identified. Morphologically, *N. coffeae* differs from closely related species *N. edgeworthiae* by having shorter conidiophores, longer conidiogenous cells, and smaller, multi-septate conidia (vs. aseptate in *N. edgeworthiae*), while *N. puerensis* differs from its close relative *N. dehongensis* by having narrower and distinctly multi-septate conidia, more tightly coiled conidial filaments, and unbranched conidiophores. These results expand the known diversity of *Neohelicomyces* and contribute to a better understanding of fungal assemblages associated with coffee plants in subtropical China. In addition, detailed descriptions, illustrations, and phylogenetic trees are provided.

## Introduction

Yunnan Province in southwestern China is a global biodiversity hotspot, supporting an exceptionally high diversity of plants and fungi (Hyde et al. 2020; Qian et al. 2020; Shen et al. 2022; Zhu and Tan 2022; Hongsanan et al. 2023). Among its major crops, coffee (*Coffea arabica* L.) is particularly important, with Yunnan accounting for nearly all of China’s coffee production (Wang et al. 2025). However, coffee plants are highly susceptible to colonization by diverse fungi, which can function as pathogens, endophytes, or saprobes, thereby influencing plant health, yield, and ecological dynamics (McCook and Vandermeer 2015; Hyde et al. 2020; Lu et al. 2022a, 2025a, 2025b). Recent surveys have revealed rich fungal assemblages associated with coffee in Yunnan, highlighting both the ecological uniqueness of the region and the economic significance of the crop (Lu et al. 2025a, 2025b; Wang et al. 2025). These factors underscore the need for continuous investigations of coffee-associated fungi in Yunnan to better understand fungal diversity and its implications for sustainable coffee production (Lu et al. 2025a, 2025b).

Tubeufiales was established by Boonmee et al. (2014) to accommodate Tubeufiaceae, which molecular phylogenetic analyses demonstrated to represent an independent lineage within Dothideomycetes, distinct from previously established orders. The circumscription of the order was supported by both morphological features and multigene phylogenies incorporating LSU, ITS, and *tef*1-α sequences (Boonmee et al. 2014). At present, Tubeufiales comprises three families: Tubeufiaceae, the type and largest family originally delineated by Barr (1979), along with Bezerromycetaceae and Wiesneriomycetaceae, which were subsequently established based on divergence-time estimates and molecular phylogenetic evidence by Liu et al. (2017). Members of the order are cosmopolitan in distribution, occurring across tropical, subtropical, and temperate regions, where they function predominantly as saprobes on decaying woody substrates in both terrestrial and freshwater habitats, particularly in aquatic environments such as streams, lakes, and swamps (Boonmee et al. 2014; Liu et al. 2017; Lu et al. 2018b; Ma et al. 2024a, 2024b; Sun et al. 2025).

The family Tubeufiaceae is a significant group within the fungal kingdom, which was established by Barr (1979) based on the generic type *Tubeufia*. This family was created to accommodate a group of fungi that exhibit both sexual and asexual morphs, with the asexual forms predominantly being helicosporous hyphomycetes (Lu et al. 2018b; Dong et al. 2020), while the sexual morph is characterized by superficial ascomata, pseudoparaphysate hamathecium, and multiseptate, hyaline to pale brown cylindrical ascospores (Barr 1980; Kodsueb et al. 2006; Boonmee et al. 2011, 2014; Brahmanage et al. 2017; Lu et al. 2018b; Ma et al. 2024a, 2024b; Peng et al. 2025; Sun et al. 2025). Members of the Tubeufiaceae are predominantly saprobic, decomposing wood and other organic matter in both terrestrial and freshwater habitats (Lu et al. 2018b; Ma et al. 2024a, 2024b; Ma et al. 2025). They are widely distributed across tropical and subtropical zones (Cai et al. 2002; Liu et al. 2018; Lu et al. 2018a, 2018b). Tubeufiaceae has been the subject of numerous taxonomic revisions and expansions in recent years. Lu et al. (2018b) provided an updated phylogenetic tree for Tubeufiales, which included 13 genera, significantly expanding the circumscription of the type family Tubeufiaceae. Subsequently, the family has been continuously refined through subsequent research, with the introduction of new genera, such as *Neomanoharachariella* (Li et al. 2022) and *Pseudotubeufia* (Ma et al. 2023). As of now, Tubeufiaceae comprises 47 genera, including well-known genera such as *Tubeufia*, *Helicoma*, *Helicosporium*, and *Neohelicomyces* (Hyde et al. 2024).

*Neohelicomyces* was established by Luo et al. (2017) based on morphological characteristics and phylogenetic analyses of combined ITS, LSU, and *tef*-1α sequence data, with *N. aquaticus* as the type species, and the genus comprises helicosporous hyphomycetes defined by coiled and helical conidia (Luo et al. 2017; Lu et al. 2018b, 2022b; Tibpromma et al. 2018; Crous et al. 2019a, 2019b; Dong et al. 2020; Hsieh et al. 2021; Yang et al. 2023; Ma et al. 2024a, 2024b). Morphologically, species of *Neohelicomyces* are characterized by macronematous, mononematous, erect, septate conidiophores, usually fertile in the lower half and sterile towards the apex, monoblastic, holoblastic, and integrated conidiogenous cells bearing lateral minute denticles that produce a single conidium each, with helicoid, multiseptated conidia, smooth-walled, pale brown, guttulate, with filaments that are tightly to loosely coiled (2–2.5 times), rounded at both ends (Luo et al. 2017; Lu et al. 2018b, 2022b; Tibpromma et al. 2018; Crous et al. 2019a, 2019b; Dong et al. 2020; Hsieh et al. 2021; Yang et al. 2023; Ma et al. 2024a, 2024b). Morphologically, *Neohelicomyces* is similar to *Helicomyces*, *Helicosporium*, and *Neohelicosporium*, but it differs from *Helicomyces* by possessing elongate, erect, and conspicuous conidiophores, and can be distinguished from *Helicosporium* by differences in conidial coiling ratio and size, and differs from *Neohelicosporium* by macronematous, mononematous, erect, septate and often conspicuous conidiophores arising directly on the substrate, with conidiogenous cells that are integrated and denticulate, and helicoid conidia that are relatively large and coiled (Tsui et al. 2006; Luo et al. 2017; Lu et al. 2018a).

In this study, fruiting bodies of two helicosporous taxa were collected and isolated from dead coffee branches in Pu’er City, Yunnan Province, China. Based on morphological characteristics, illustrations, and multi-gene phylogenetic analyses, the two taxa were identified as novel species within *Neohelicomyces*, namely *N. coffeae* and *N. puerensis*.

## Materials and methods

### Specimen collection, examination, and isolation

Pu’er City is one of the major coffee-growing regions in China, characterized by a warm and humid subtropical monsoon climate that provides favorable conditions for both coffee cultivation and fungal diversity (Zhang et al. 2020). In this study, sampling targeted decaying branches of *C. arabica* bearing white fungal fruiting bodies in Pu’er City, Yunnan Province, China. Samples were placed in self-sealing plastic bags, with detailed collection data recorded in the field (Rathnayaka et al. 2025) and subsequently transported to the mycology laboratory at Qujing Normal University, China. The external features of fruiting bodies on specimen surfaces were first examined using a stereomicroscope (OLYMPUS SZ61, Japan). Micromorphological features were observed using a compound microscope (OLYMPUS BX53, Olympus, Tokyo, Japan), and images were captured with an OLYMPUS DP74-CU digital camera. Measurements were performed using Tarosoft® Image Framework v. 0.9.7 software, and final photographic plates were prepared using Adobe Photoshop CC 2019 v. 23.1.0 (Adobe Systems, USA).

Pure cultures were isolated through single-spore isolation, following the procedures described by Senanayake et al. (2020). Germinated conidia were transferred to fresh potato dextrose agar (PDA) plates and incubated at 28 °C for 2–6 weeks, after which colony morphology was examined. Dried herbarium specimens were preserved in the Herbarium of Guizhou Medical University (GMB-W, Guiyang, China), while living cultures were deposited in the Guizhou Medical University Culture Collection (GMBCC, Guiyang). Index Fungorum (IF) numbers were assigned following Index Fungorum (2026).

### DNA extraction, PCR amplification, and sequencing

Fresh mycelia were scraped from the surface of colonies (2–6 weeks grown on PDA) with a sterilized toothpick and transferred to a 1.5 ml microcentrifuge tube for DNA extraction. Genomic DNA was extracted using the Biospin Fungus Genomic DNA Extraction Kit-BSC14S1 (BioFlux, China), following the manufacturer’s protocol. Primer pairs ITS5/ITS4 (White et al. 1990), LR0R/LR5 (Vilgalys and Hester 1990), EF1-983F/EF1-2218R (Rehner and Samuels 1994), and fRPB2-5F/fRPB2-7cR (Liu et al. 1999) were used to amplify ITS, LSU, *tef*1-α, and *rpb*2 sequence fragments, respectively. The PCR reactions were carried out in a total volume of 25 µL, containing 12.5 µL of 2 × Bench Top™ Taq Master Mix, 8.5 µL of ddH_2_O, 1 µL of each forward and reverse primer, and 2 µL of DNA template. PCR amplification of the ITS, LSU, and *tef*1-α regions was performed under the following conditions: an initial denaturation at 94 °C for 3 min, followed by 35 cycles of denaturation at 94 °C for 45 s, annealing at 55 °C for 50 s, and extension at 72 °C for 60 s, with a final extension at 72 °C for 10 min. For *rpb*2, PCR was conducted under similar conditions, except for an initial denaturation at 95 °C for 5 min, followed by 40 cycles of 95 °C for 1 min, 55 °C for 2 min, and 72 °C for 90 s, with a final extension at 72 °C for 10 min. The PCR products were purified and sequenced by Sangon Biotechnology Co., Ltd. (Shanghai, China). All new sequences generated in this study were submitted to GenBank (http://www.ncbi.nlm.nih.gov/genbank/) and are detailed in Table 1.

**Table 1.** Taxa used in this study, along with their corresponding GenBank accession numbers. The newly generated sequences are in bold black. After the strain number, “^T^” indicates the type strains. “NA” indicates sequence unavailability.

Fungal species	Strain numbers	ITS	LSU	*tef*1-α	*rpb*2
*Bezerromyces brasiliensis*	CBS 141545	NR_153463	NG_069376	NA	NA
*Bezerromyces brasiliensis*	URM7411	KX470390	KX518623	KX518631	NA
*Muripulchra aquatica*	DLUCC 0571	KY320531	KY320548	NA	NA
*Muripulchra aquatica*	KUMCC 15-0245	KY320533	KY320550	KY320563	MH551057
*Muripulchra aquatica*	KUMCC 15-0276	KY320534	KY320551	KY320564	MH551058
*Muripulchra aquatica*	MFLUCC 15-0249^T^	KY320532	KY320549	NA	NA
*Neohelicomyces acropleurogenus*	CGMCC 3.25549^T^	PP626594	PP639450	PP596351	PP596478
*Neohelicomyces aquaticus*	KUMCC 15-0463	KY320529	KY320546	KY320562	MH551065
*Neohelicomyces aquaticus*	MFLUCC 16-0993^T^	KY320528	KY320545	KY320561	MH551066
*Neohelicomyces aquisubtropicus*	GZCC 23-0080^T^	PQ098499	PQ098537	PV768327	PV768336
*Neohelicomyces aquisubtropicus*	GZCC 24-0163	PV730410	PV730414	PV768328	PV768337
*Neohelicomyces aseptatus*	CGMCC 3.25564^T^	PP626595	PP639451	PP596352	PP596479
*Neohelicomyces coffeae*	GMBCC 2225^T^	PX308843	PX308848	PX314510	PX314514
*Neohelicomyces coffeae*	GMBCC 2226	PX308844	PX308849	PX314511	PX314515
*Neohelicomyces dehongensis*	MFLUCC 18-1029^T^	NR_171880	MN913709	MT954393	NA
*Neohelicomyces denticulatus*	GZCC 19-0444^T^	OP377832	MW133855	NA	NA
*Neohelicomyces denticulatus*	UAMH 10535	AY916462	AY856913	NA	NA
*Neohelicomyces denticulatus*	GZCC 23-0073^T^	PP626596	PP639452	PP596353	PP596480
*Neohelicomyces deschampsiae*	CPC 33686^T^	MK442602	MK442538	NA	NA
*Neohelicomyces edgeworthiae*	CGMCC 3.25565^T^	PP626597	PP639453	PP596354	PP596481
*Neohelicomyces grandisporus*	KUMCC 15-0470^T^	KX454173	KX454174	NA	MH551067
*Neohelicomyces guizhouensis*	GZCC 23-0725^T^	PP512969	PP512973	PP526727	PP526733
*Neohelicomyces guizhouensis*	GZCC 23-0726	PP512970	PP512974	PP526728	PP526734
*Neohelicomyces guttulatus*	CGMCC 3.25550^T^	PP626598	PP639454	PP596355	NA
*Neohelicomyces guttulatus*	GZCC 23-0406	PP626599	PP639455	PP596356	PP596482
*Neohelicomyces hainanensis*	GZCC 22-2009^T^	OP508734	OP508774	OP698085	OP698074
*Neohelicomyces hainanensis*	GZCC 22-2027	OP508735	OP508775	OP698086	OP698075
*Neohelicomyces helicosporus*	GZCC 23-0633^T^	PP512971	PP512975	PP526729	PP526735
*Neohelicomyces helicosporus*	GZCC 23-0634	PP512972	PP512976	PP526730	PP526736
*Neohelicomyces hyalosporus*	GZCC 16-0086^T^	MH558745	MH558870	MH550936	MH551064
*Neohelicomyces hydei*	GZCC 23-0727^T^	NA	PP512977	PP526731	PP526737
*Neohelicomyces hydei*	GZCC 23-0728	NA	PP512978	PP526732	PP526738
*Neohelicomyces lignicola*	CGMCC 3.25551^T^	PP626600	PP639456	PP596357	PP596483
*Neohelicomyces longisetosus*	NCYU 106H1-1-1^T^	MT939303	NA	NA	NA
*Neohelicomyces macrosporus*	CGMCC 3.25552^T^	PP626601	PP639457	PP596358	PP596484
*Neohelicomyces maolanensis*	GZCC 23-0079^T^	NA	PQ098529	PQ490683	PQ490677
*Neohelicomyces maolanensis*	GZCC 24-0148	NA	PQ522500	PQ490682	PQ490676
*Neohelicomyces melaleucae*	CPC 38042^T^	MN562154	MN567661	MN556835	NA
*Neohelicomyces melaleucae*	KUNCC 23-14314	PP664108	PP664112	NA	NA
*Neohelicomyces pallidus*	CBS 245.49	MH856510	NA	NA	NA
*Neohelicomyces pallidus*	CBS 271.52	AY916461	AY856887	NA	NA
*Neohelicomyces pallidus*	CBS 962.69	AY916460	AY856886	NA	NA
*Neohelicomyces pandanicola*	KUMCC 16-0143^T^	MH275073	MH260307	MH412779	NA
*Neohelicomyces puerensis*	GMBCC 2217^T^	PQ737369	PX308846	PX314508	PX314512
*Neohelicomyces puerensis*	GMBCC 2218	PQ737370	PX308847	PX314509	PX314513
*Neohelicomyces qixingyaensis*	CGMCC 3.25569^T^	PP626602	PP639458	PP596359	PP596485
*Neohelicomyces sexualis*	HGUP 24-0021	PQ570844	PQ570861	NA	NA
*Neohelicomyces subtropicus*	GZCC 23-0076^T^	PQ098492	PQ098530	PQ490685	PQ490679
*Neohelicomyces subtropicus*	GZCC 24-0147	PQ522498	PQ522501	PQ490684	PQ490678
*Neohelicomyces thailandicus*	MFLUCC 11-0005^T^	NR_171882	MN913696	NA	NA
*Neohelicomyces thailandicus*	GZCC 23-0400	PP626603	PP639459	PP596360	PP596486
*Neohelicomyces submersus*	MFLUCC 16-1106^T^	KY320530	KY320547	NA	MH551068
*Neohelicomyces wuzhishanensis*	GZCC 23-0410^T^	PQ098494	PQ098532	PV768325	PV768334
*Neohelicomyces wuzhishanensis*	GZCC 24-0164	PV730409	PV730413	PV768326	PV768335
*Neohelicomyces xiayadongensis*	CGMCC 3.25568^T^	PP626604	PP639460	PP596361	PP596487
*Neohelicomyces xiayadongensis*	MUCL 15702	AY916459	AY856873	NA	NA
*Neohelicomyces yunnanensis*	GZCC 23-0735^T^	PP664109	PP664113	NA	NA
*Neohelicosporium bambusicola*	MFLUCC 21-0156^T^	OL606157	OL606146	OL964517	OL964523
*Neohelicosporium ellipsoideum*	MFLUCC 16-0229^T^	MH558748	MH558873	MH550939	MH551071
*Neohelicosporium fusisporum*	MFUCC 16-0642^T^	MG017612	MG017613	MG017614	NA
*Neohelicosporium guineensis*	ZHKUCC 24-0113^T^	PP860090	PP860102	PP858062	PP858074
*Neohelicosporium hyalosporum*	GZCC 16-0076^T^	MF467923	MF467936	MF535249	MF535279
*Neohelicosporium irregulare*	MFLUCC 17-1796^T^	MH558752	MH558877	MH550943	MH551075
*Neohelicosporium krabiense*	MFLUCC 16-0224^T^	MH558754	MH558879	MH550945	MH551077
*Neohelicosporium laxisporum*	MFLUCC 17-2027^T^	MH558755	MH558880	MH550946	MH551078
*Tubeufia guttulata*	GZCC 23-0404^T^	OR030841	OR030834	OR046678	OR046684
*Tubeufia guttulata*	GZCC 23-0590	OR066413	OR066420	OR058859	OR058852
*Tubeufia hainanensis*	GZCC 22-2015^T^	OR030842	OR030835	OR046679	OR046685
*Tubeufia javanica*	MFLUCC 12-0545^T^	KJ880034	KJ880036	KJ880037	NA
*Tubeufia krabiensis*	MFLUCC 16-0228^T^	MH558792	MH558917	MH550985	MH551118
*Tubeufia latispora*	MFLUCC 16-0027^T^	KY092417	KY092412	KY117033	MH551119
*Tubeufia laxispora*	MFLUCC 16-0232^T^	KY092413	KY092408	KY117029	MF535287
*Tubeufia machaerinae*	MFLUCC 17-0055	MH558795	MH558920	MH550988	MH551122
*Tubeufia mackenziei*	MFLUCC 16-0222^T^	KY092415	KY092410	KY117031	MF535288
*Tubeufia muriformis*	GZCC 22-2039^T^	OR030843	OR030836	OR046680	OR046686
*Tubeufia nigroseptum*	CGMCC 3.20430^T^	MZ092716	MZ853187	OM022002	OM022001
*Tubeufia pandanicola*	MFLUCC 16-0321^T^	MH275091	MH260325	NA	NA

### Phylogenetic analyses

Raw forward and reverse reads generated in this study were first checked for quality and assembled using BioEdit v. 7.0.5.3 (Hall 1999) and SeqMan v. 7.0.0 (DNASTAR, Madison, WI, USA) (Swindell and Plasterer 1997). The sequences incorporated in this study were downloaded from GenBank (https://www.ncbi.nlm.nih.gov). Each locus was individually aligned using the MAFFT v.7 online server (https://mafft.cbrc.jp/alignment/server/) (Katoh et al. 2019), and poorly aligned or ambiguous regions were removed with trimAl v. 1.2 (Capella-Gutiérrez et al. 2009), followed by manual adjustments in BioEdit v. 7.0.5.3. The multilocus datasets (ITS+LSU+*rpb*2+*tef*1-α) were concatenated in SequenceMatrix v. 1.7.8 (Vaidya et al. 2011). Maximum likelihood (ML) and Bayesian inference (BI) analyses were carried out on the CIPRES Science Gateway platform (https://www.phylo.org/portal2/home.action) (Dissanayake et al. 2020). The ML tree was inferred using RAxML-HPC v.8 on XSEDE (Stamatakis 2006), applying the GTRGAMMA model with 1,000 bootstrap replicates. BI tree was implemented in MrBayes v. 3.2.7a on XSEDE (Huelsenbeck and Ronquist 2001; Ronquist et al. 2012), under the Markov Chain Monte Carlo (MCMC) framework, with six chains running for 2,000,000 generations and trees sampled every 100^th^ generation. The multi-gene phylogenetic trees were visualized using FigTree v. 1.4.2 (http://tree.bio.ed.ac.uk/software/figtree/) and refined in Adobe Illustrator CS6 Adobe Systems Inc., USA).

## Results

### Phylogenetic analysis results

**Figure 1.**
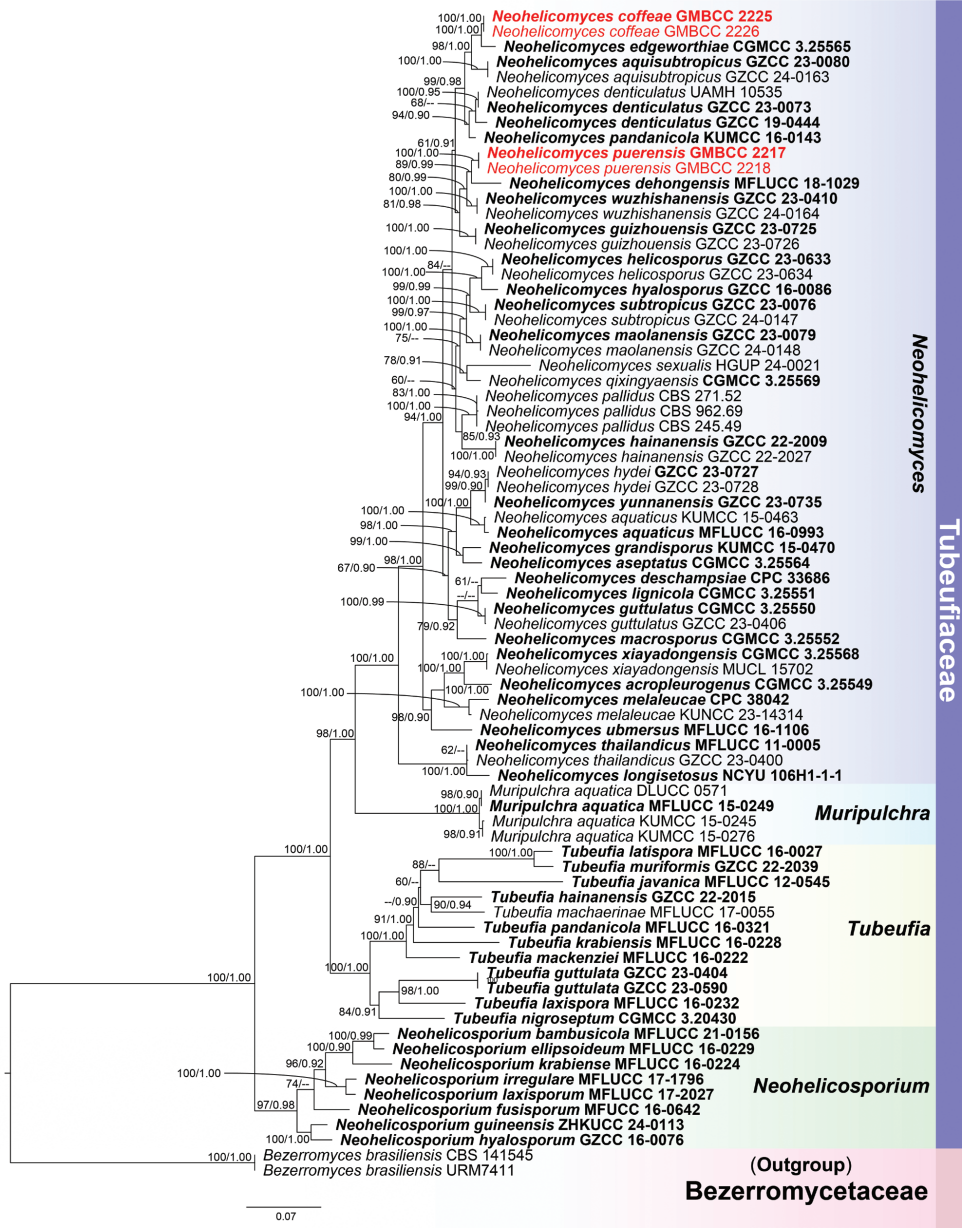
Phylogenetic tree generated from Maximum Likelihood (ML) analysis, based on the combined ITS+LSU+*rpb*2+*tef*1-α sequence data. Bootstrap support values of ML equal to or greater than 60% and Bayesian posterior probabilities (PP) equal to or greater than 0.90 are given near the nodes as ML/PP, respectively. The new species are typed in bold red, while the ex-type strains are in red.

The phylogenetic positions of our newly introduced species were determined based on multi-gene (ITS+LSU+*rpb*2+*tef*1-α) phylogenetic analysis. The concatenated sequence matrix comprised 3,126 characters (ITS: 1–597, LSU: 598–1,386, *rpb*2: 1,387–2,273, and *tef*1-α: 2,274–3,126) across 75 ingroup and two outgroup taxa, *Bezerromyces brasiliensis* (CBS 141545 and URM7411). Both the ML and BI analyses of the concatenated ITS, LSU, *tef*1-α, and *rpb*2 datasets yielded similar tree topologies. The best-scoring RAxML tree, with a final ML optimization likelihood value of -22094.627605, is presented. The matrix contained 1064 distinct alignment patterns, with 21.45% of the characters being undetermined or gaps. Estimated base frequencies were as follows: A = 0.247173, C = 0.247452, G = 0.260954, T = 0.244421; substitution rates: AC = 0.918770, AG = 4.818137, AT = 2.150864, CG = 0.889941, CT = 8.655879, GT = 1.000000; gamma distribution shape parameter α = 0.176011. The phylogenetic tree resulting from RAxML analysis is shown in Fig. 1. With reference to the multi-gene phylogram (Fig. 1), our collections represent two distinct *Neohelicomyces* species within Tubeufiaceae. Our isolates, *N. coffeae* (GMBCC 2225 and GMBCC 2226), cluster together with *N. edgeworthiae* (CGMCC 3.25565) as a sister lineage receiving 100% ML/1.00 PP support. Two additional strains from our collections, *N. puerensis* (GMBCC 2217 and GMBCC 2218), belong to a sister lineage to *N. dehongensis* (MFLUCC 18-1029), with 89% ML/0.99 PP support. Comparisons of nucleotide sequences showed that *N. coffeae* and *N. edgeworthiae* differed by 14/550 bp (2.54%, 5 gaps) in ITS, 1/913 bp (0.1%, no gap) in LSU, 7/924 bp (0.86%, 1 gap) in *tef*1-α, and 15/1,009 bp (1.48%, 13 gaps) in *rpb*2. Likewise, *N. puerensis* and *N. dehongensis* differed by 34/933 bp (3.6%, no gaps) in ITS, 7/870 bp (0.8%, no gaps) in LSU, and 16/887 bp (1.8%, no gaps) in *tef*1-α.

The phylogenetic tree (Fig. 1) clearly resolves *Neohelicomyces* as a strongly supported monophyletic clade within Tubeufiaceae (98% ML/1.00 PP). All species of *Neohelicomyces* are well separated with statistical support, and several well-defined subclades are recovered, such as the grouping of *N. edgeworthiae* with *N. aquisubtropicus* and *N. denticulatus* with *N. pandanicola*. The related genera *Muriuplucha*, *Tubeufia*, and *Neohelicosporium* are also recovered as distinct, well-supported lineages, clearly separated from *Neohelicomyces*.

### Taxonomy

#### Neohelicomyces coffeae

Taxon classificationFungiTubeufialesTubeufiaceae

M.Y. Han & S. Tibpromma sp. nov.

0051396C-B4DB-54B6-9705-4258973BBA0A

Index Fungorum: IF904424

Fig. 2

##### Etymology.

The species epithet “coffeae” refers to the host genus *Coffea*.

##### Holotype.

GMB-W1490.

##### Description.

***Saprobic*** on dead branches of *C. arabica*. ***Sexual morph***: Undetermined. ***Asexual morph***: Hyphomycetous, helicosporous. ***Colonies*** on natural substrate superficial, effuse, gregarious, white, can be seen as a white bush with the naked eye. ***Mycelium*** partly immersed to superficial, composed of hyaline to pale brown, branched, septate, smooth hyphae. ***Colonies*** composed of numerous coiled, spirally twisted, hyaline conidia aggregated in fascicles, which reflect light and impart a glistening, sparkling appearance to the colony surface. ***Conidiophores*** 144.5–274 × 3.4–6.2 μm (x̄ = 201 × 4.6 μm, *n* = 30), macronematous, mononematous, erect or sub-erect, produced singly or in groups, some arising from creeping hyphae, cylindrical, straight or slightly flexuous, occasionally branched, septate, subhyaline to pale brown, thick-walled. ***Conidiogenous cells*** 13.2–20.6 × 3.4–5.2 μm (x̄ = 17 × 4.1 μm, *n* = 25), holoblastic, monoblastic or polyblastic, integrated, terminal or intercalary, cylindrical, with denticles, hyaline, smooth-walled, truncate at apex after conidial secession, subhyaline to pale brown, smooth-walled. ***Conidia*** solitary, acropleurogenous, helicoid, tapering towards the rounded ends, developing on a tooth-like protrusion, 13.5–19.4 μm diam. (x̄ = 17.1 μm, *n* = 25) and conidial filament 2.1–3.3 μm wide (x̄ = 2.8 μm, *n* = 25), 105.3–146.4 μm long (x̄ = 132 μm, *n* = 25), tightly coiled 2.5–3.75 times, loosely coiled three times, becoming loosely coiled in water, multi-septate, hyaline to subhyaline, some in pale brown, smooth-walled, granules.

**Figure 2.**
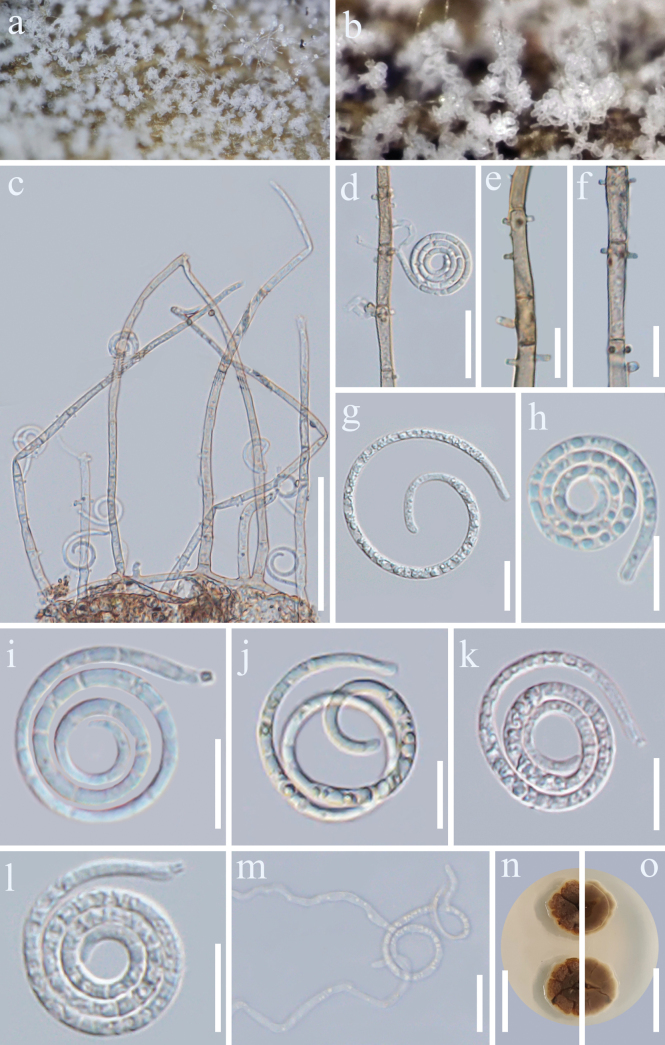
*Neohelicomyces coffeae* (GMB-W1490, holotype). **a, b**. Colonies on the host surface; **c**. Conidiophores, conidiogenous cells and conidia; **d**. Conidiogenous cells and conidia; **e, f**. Conidiogenous cells; **g–l**. Conidia; **m**. Germinated conidium; **n, o**. Surface and reverse colonies on PDA; **n** is the surface side, **o** is the reverse sid**e**. Scale bars: 50 μm (**c**); 25 mm (**n, o**); 20 μm (**d, m**); 10 μm (**e–l**).

##### Culture characteristics.

Conidia germinated on PDA and produced germ tubes within 12 h. Colonies on PDA reaching 22 mm diam. after two weeks of incubation at 28 °C with an irregular shape, rough surface, slightly raised, radially striated with a lobate edge. Colony surface centrally dark brown to chestnut-brown, becoming paler towards the periphery; aerial mycelium sparse to moderate, whitish at the margin. Reverse side uniformly dark brown.

##### Material examined.

China • Yunnan Province, Pu’er City, Simao District (22°36'36"N, 101°0'14"E, 1189 m), on dead branches of *C. arabica* L., 1 August 2024, M.Y. Han, (BG55 = GMB-W1490, holotype), ex-type GMBCC 2225, other living culture GMBCC 2226.

##### GenBank number.

GMBCC 2225 = ITS: PX308843, LSU: PX308848, *tef*1-α:PX314510, *rpb*2: PX314514 and GMBCC 2226 = ITS: PX308844, LSU: PX308849, *tef*1-α: PX314511, *rpb*2: PX314515.

##### Notes.

In the phylogenetic analyses (Fig. 1), our collections of *N. coffeae* [GMBCC 2225 (ex-type) and GMBCC 2226] clustered with *N. edgeworthiae* (CGMCC 3.25565) as a sister lineage, receiving 100% ML/1.00 PP support. Comparisons of nucleotide sequences between *N. coffeae* and *N. edgeworthiae* revealed minor overall differences; however, the ITS region showed a 14/550 bp (2.54%, 5 gaps) difference. Morphologically, the conidiogenous cells of our isolates are notably longer than those of *N. edgeworthiae* (13.2–20.6 μm vs. 9–14 μm) (Ma et al. 2024b). Conversely, the conidia of our collections are significantly smaller than those of *N. edgeworthus*, both in diameter (13.5–19.4 μm vs. 21.5–34 μm) and length (105.3–146.4 μm vs. 121–177 μm) (Ma et al. 2024b). In addition, the conidia of *N. edgeworthiae* are aseptate and have numerous guttulate, whereas conidia of our collections are multi-septate, lack guttulate, and have only surface granules (Ma et al. 2024b). In summary, although the nucleotide divergence between our collections and *N. edgeworthiae* is relatively low, the combination of distinct morphological characters and phylogenetic position supports their separation, and therefore, we introduce *N. coffeae* herein as a novel species.

#### Neohelicomyces puerensis

Taxon classificationFungiTubeufialesTubeufiaceae

M.Y. Han & Tibpromma S. sp. nov.

BFC4DBB2-7269-56E3-9D0D-EF7CAA1DF804

Index Fungorum: IF904425

Fig. 3

##### Etymology.

The species name “puerensis” refers to the type locality, Pu’er City, China.

##### Holotype.

GMB-W1485.

##### Description.

***Saprobic*** on dead branches of *C. arabica*. Sexual morph Undetermined. ***Asexual morph*** Hyphomycetous, helicosporous. ***Colonies*** on natural substrate superficial, effuse, gregarious, white, can be seen as a white bush with the naked eye. ***Mycelium*** partly immersed, partly superficial, composed of hyaline to pale brown, branched, septate, smooth hyphae, comprising glistening conidial mass. ***Conidiophores*** 138.8–211.7 × 2.6–4.2 μm (x̄ = 182.4 × 3.4 μm, *n* = 30), macronematous, mononematous, some arising from creeping hyphae, erect, cylindrical, straight or slightly flexuous, unbranched or occasionally branched, septate, pale brown to brown at base, paler towards the apex, thick-walled. ***Conidiogenous cells*** 11.6–20 × 2.4–4.1 μm (x̄ = 14.8 × 3.2 μm, *n* = 25), holoblastic, monoblastic or polyblastic, integrated, terminal or intercalary, cylindrical, with denticles, hyaline to pale brown, smooth-walled. ***Conidia*** solitary, acropleurogenous, helicoid, tapering towards the rounded ends, developing on a tooth-like protrusion, 15.2–22 μm diam. (x̄ = 17.94 μm, *n* = 25) and conidial filament 1.9–3.7 μm wide (x̄ = 2.5 μm, *n* = 20), 106.6–211.2 μm long (x̄ = 138 μm, *n* = 30), tightly coiled 2.5–4.25 times, becoming loosely coiled in water, multi-septate, guttulate, hyaline, smooth-walled.

**Figure 3.**
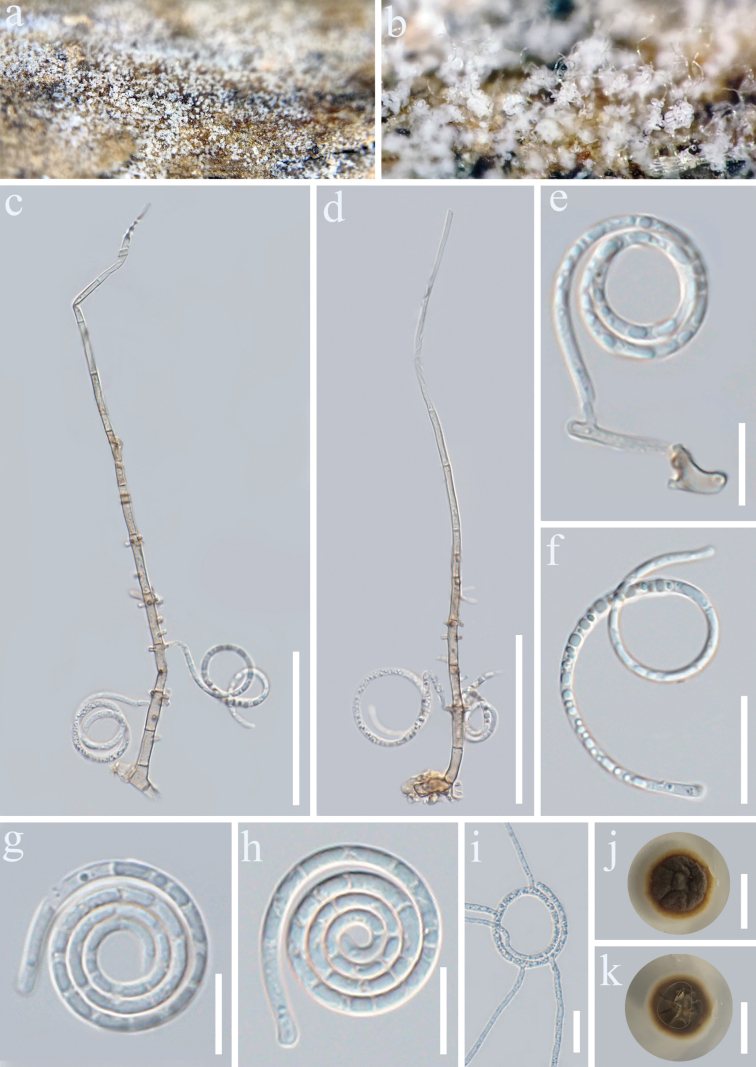
*Neohelicomyces puerensis* (GMB-W1485, holotype). **a, b**. Colonies on the host surface; **c, d**. Conidiophores, conidiogenous cells, and conidia; **e**. Conidiogenous cells and conidia; **f–h**. Conidia; **i**. Cerminated conidium; **j, k**. Surface and reverse colonies on PDA. Scale bars: 50 μm (**c, d**); 25 mm (**j, k**), 20 μm (**f, i**), 10 μm (**e, g, h**).

##### Culture characteristics.

Conidia germinated on PDA and produced germ tubes within 10 h. Colonies on PDA reaching 32 mm diam. after 30 days of incubation at 25 °C. The colony is regularly circular in shape, with a convex surface; the central part is cracked, resembling a petal-like appearance, dark gray to brownish, with a pale brown margin. Reverse side is dark brown, with the margin varying from brown to white.

##### Material examined.

China • Yunnan Province, Pu’er City, Simao District (22°36'36"N, 101°0'14"E, 1189 m), on dead branches of *C. arabica*, 1 August 2024, M.Y. Han, (BG21 = GMB-W1485, holotype), ex-type GMBCC 2217, other living culture GMBCC 2218.

##### GenBank number.

GMBCC 2217 = ITS: PQ737369, LSU: PX308846, *tef*1-α: PX314508, *rpb*2: PX314512 and GMBCC 2218 = ITS: PQ737370, LSU: PX308847, *tef*1-α: PX314509, *rpb*2: PX314513.

##### Notes.

In the concatenated phylogenetic analysis, our strains [GMBCC 2217 (ex-type) and GMBCC 2218] of *N. puerensis* formed a sister branch to *N. dehongensis* (MFLUCC 18-1029) with 89% ML/0.99 PP statistical support (Fig. 1). Sequence comparisons between *N. coffeae* and *N. dehongensis* revealed greater divergence in the ITS region (34/933 bp; 3.6%, no gap). Morphologically, our species resembles *N. dehongensis* in having macronematous and mononematous conidiophores, holoblastic conidiogenous cells with denticles, and helicoid, multi-septate, hyaline conidia (Dong et al. 2020). However, it differs from *N. dehongensis* in having slightly shorter conidiophores (138.8–211.7 µm vs. 120–250 µm) and conidia (106.6–211.2 µm vs. 145–210 µm), as well as narrower conidial diameters (15.2–22 µm vs. 20–25 µm) (Dong et al. 2020). In addition, conidia of *N. puerensis* are distinctly multi-septate, whereas those of *N. dehongensis* are indistinctly septate. The conidial filaments in our collections are more tightly coiled (2.5–4.25 times vs. 2.75–3.75 times), and conidiophores are usually unbranched, lacking the basal forking that is frequently observed in *N. dehongensis*. Moreover, conidiogenous cells of *N. puerensis* are terminal or intercalary, whereas those of *N. dehongensis* are mostly intercalary and sympodial. Thus, based on these morphological differences and molecular evidence, we propose that *N. puerensis* be recognized as a new species.

## Discussion

Coffee ecosystems host diverse fungal assemblages, including pathogens, endophytes, and saprophytes (McCook and Vandermeer 2015; Lu et al. 2022a, 2025a, 2025b). Although saprophytic fungi account for a relatively small proportion of these communities (Lu et al. 2022a, 2025a, 2025b), they play critical roles in litter decomposition, nutrient cycling, and maintaining ecosystem stability (Liu et al. 2022; Zhang et al. 2023a). Recent surveys of coffee agroecosystems have revealed a rapid increase in previously unreported saprophytic fungi, with the known record expanding to more than 150 species, including numerous new taxa and new records (Lu et al. 2022a, 2025a, 2025b; Han et al. 2024, 2025a, 2025b).

Beyond their ecological functions, many saprophytic fungi are also recognized as promising sources of bioactive metabolites with potential applications in medicine and agriculture (Bills and Gloer 2016; Lu et al. 2018b; Keller 2019; Zhang et al. 2024). In particular, members of the family Tubeufiaceae have been reported to produce a range of bioactive secondary metabolites with antifungal, antibacterial, antidiabetic, and anticancer activities (Itazaki et al. 1990; Hanada et al. 1996; Hu et al. 2006; Lu et al. 2018b; Zhang et al. 2023b, 2024). Within this family, taxonomic studies of *Neohelicomyces* have not only clarified species diversity and distribution, but also highlighted their chemical potential (Zheng et al. 2023; Ma et al. 2024a, 2024b). For example, compounds isolated from *N. hyalosporus* (PF11-1) exhibited moderate cytotoxicity against human cancer cell lines, including A549, TCA, and RD (Zheng et al. 2023). These findings suggest that *Neohelicomyces* could provide valuable candidate strains for the development of oncology-related drugs.

According to Index Fungorum (2026), *Neohelicomyces* currently comprises 36 species distributed across Asia (especially China (35/36 = 97.22%), with Guizhou Province as a diversity hotspot, following by Yunnan, Guangxi, Hainan, Tibet, Guangdong, and other regions), Europe (2/36 = 5.56%), and North America (2/36 = 5.56%) (Note: percentages are calculated as the proportion of the 36 accepted species that have been reported from each region; species occurring in multiple regions were counted in each relevant region) (Luo et al. 2017; Tibpromma et al. 2018; Crous et al. 2019a, 2019b; Dong et al. 2020; Hsieh et al. 2021; Lu et al. 2022b; Yang et al. 2023; Ma et al. 2024a, 2024b, Ma et al. 2025; Peng et al. 2025; Sun et al. 2025). Most species inhabit freshwater driftwood or moist substrates, e.g., *N. aquaticus*, *N. grandisporus*, *N. submersus* (Luo et al. 2017), *N. thailandicus* (Dong et al. 2020), *N. hydei*, *N. lignicola*, *N. macrosporus* (Ma et al. 2024a, 2024b), while others occur on terrestrial plant tissues, e.g., *N. melaleucae* on leaves (Crous et al. 2019b), *N. pandanicola* on roots (Tibpromma et al. 2018). In particular, *N. pallidus* has been recorded from multiple countries, including China, the Czech Republic, Italy, Japan, the Netherlands, and the United States, and exhibits both freshwater and terrestrial occurrences (Linder 1929; Goos 1989; Tsui et al. 2001; Zhao et al. 2007; Lu et al. 2018b; Ma et al. 2024b). This ecological versatility suggests strong adaptive potential.

In this study, we report two novel *Neohelicomyces* species (*N. coffeae* and *N. puerensis*) isolated from decaying branches of *C. arabica*. This discovery marks the first records of new taxa of this genus from coffee hosts, thereby extending its known host and substrate range into agricultural ecosystems. Morphologically, species of *Neohelicomyces* are generally similar, but they can be differentiated by several diagnostic features, such as the size of conidiophores and conidia, the diameter and times of coils of conidia, the presence or absence of septa in conidia, and whether conidiophores are branched. In addition to these morphological characteristics, DNA sequence data, particularly from the ITS region, provide robust evidence for species delimitation. Beyond their taxonomic significance, these species may represent candidate strains for the exploration of bioactive secondary metabolites. Furthermore, by expanding the known diversity of *Neohelicomyces* in agricultural environments, our findings provide a foundation for future studies that integrate taxonomy, ecology, and natural product research, and highlight the potential of saprophytic fungi as reservoirs of novel metabolites with pharmaceutical and biotechnological applications (Strobel and Daisy 2003; Duong et al. 2020; Zheng et al. 2023; Han et al. 2025a).

## Supplementary Material

XML Treatment for Neohelicomyces coffeae

XML Treatment for Neohelicomyces puerensis

## Additional information

### Use of AI

No use of AI was reported.

### Author ORCIDs

Mei-Yan Han https://orcid.org/0009-0004-3705-5408

Jing-Ya Yang https://orcid.org/0009-0000-0375-990X

Samantha C. Karunarathna https://orcid.org/0000-0001-7080-0781

Jaturong Kumla https://orcid.org/0000-0002-3673-6541

Li Lu https://orcid.org/0000-0003-0977-6414

De-Ge Zheng https://orcid.org/0009-0001-9199-6784

Abdallah M. Elgorban https://orcid.org/0000-0003-3664-7853

Alanoud T. Alfagham https://orcid.org/0000-0001-9008-3679

Fu-Qiang Yu https://orcid.org/0000-0001-7931-080X

Dong-Qin Dai https://orcid.org/0000-0001-8935-8807

Li-Juan Zhang https://orcid.org/0000-0002-3234-6757

Nakarin Suwannarach https://orcid.org/0000-0002-2653-1913

Saowaluck Tibpromma https://orcid.org/0000-0002-4706-6547

### Data availability

All of the data that support the findings of this study are available in the main text.
